# Bis(benzyl­aminium) 4,5-dichloro­benzene-1,2-dicarboxyl­ate monohydrate

**DOI:** 10.1107/S1600536812023458

**Published:** 2012-05-31

**Authors:** Graham Smith, Urs D. Wermuth

**Affiliations:** a Science and Engineering Faculty, Queensland University of Technology, GPO Box 2434, Brisbane, Queensland 4001, Australia

## Abstract

In the structure of the title salt, 2C_7_H_10_N^+^·C_8_H_2_Cl_2_O_4_^2−^·H_2_O, the two benzyl­aminium anions have different conformations, one being essentially planar and the other having the side chain rotated out of the benzene plane [minimum ring to side-chain C—C—C—N torsion angles = −3.6 (6) and 50.1 (5)°, respectively]. In the 4,5-dichloro­phthalate dianion, the carboxyl­ate groups make dihedral angles of 23.0 (2) and 76.5 (2)° with the benzene ring. In the crystal, aminium N—H⋯O and water O—H⋯O hydrogen-bonding associations with carboxyl­ate O-atom acceptors give a two-dimensional duplex sheet structure which extends along the (011) plane. Weak π–π inter­actions are also present between the benzene ring of the dianion and one of the cation rings [minimum ring centroid separation = 2.749 (3) Å].

## Related literature

For the crystal structures of some 1:1 Lewis base salts of 4,5-dichloro­phthalic acid, see: Mattes & Dorau (1986 ▶); Smith *et al.* (2008 ▶). For crystal structures having dianionic 4,5-dichloro­phthalate species, see: Büyükgüngör & Odabaşoğlu (2007 ▶); Smith & Wermuth (2010 ▶, 2011 ▶).
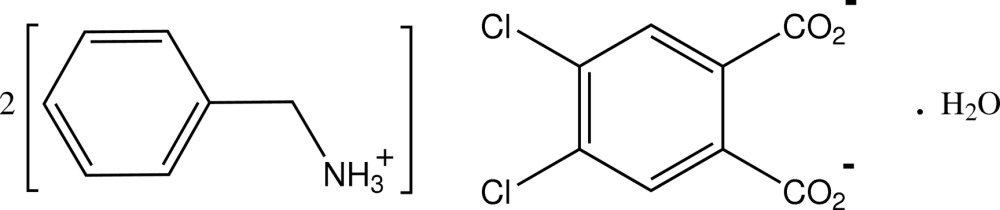


## Experimental

### 

#### Crystal data

2C_7_H_10_N^+^·C_8_H_2_Cl_2_O_4_^2−^·H_2_O
*M* *_r_* = 467.33
Monoclinic, 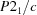

*a* = 17.3005 (16) Å
*b* = 10.0084 (7) Å
*c* = 13.6990 (12) Å
β = 112.641 (11)°
*V* = 2189.2 (4) Å^3^
*Z* = 4
Mo *K*α radiation
μ = 0.33 mm^−1^
*T* = 200 K
0.33 × 0.22 × 0.12 mm

#### Data collection

Oxford Diffraction Gemini-S CCD-detector diffractometer
Absorption correction: multi-scan (*CrysAlis PRO*; Oxford Diffraction, 2010 ▶) *T*_min_ = 0.86, *T*_max_ = 0.98
12311 measured reflections
3842 independent reflections
3019 reflections with *I* > 2σ(*I*)
*R*_int_ = 0.045

#### Refinement

*R*[*F* ^2^ > 2σ(*F* ^2^)] = 0.068
*wR*(*F* ^2^) = 0.189
*S* = 1.19
3842 reflections
312 parameters
H-atom parameters constrained
Δρ_max_ = 0.79 e Å^−3^
Δρ_min_ = −0.37 e Å^−3^

### 

Data collection: *CrysAlis PRO* (Oxford Diffraction, 2010 ▶); cell refinement: *CrysAlis PRO*; data reduction: *CrysAlis PRO*; program(s) used to solve structure: *SHELXS97* (Sheldrick, 2008 ▶); program(s) used to refine structure: *SHELXL97* (Sheldrick, 2008 ▶) within *WinGX* (Farrugia, 1999 ▶); molecular graphics: *PLATON* (Spek, 2009 ▶); software used to prepare material for publication: *PLATON*.

## Supplementary Material

Crystal structure: contains datablock(s) global, I. DOI: 10.1107/S1600536812023458/wn2476sup1.cif

Structure factors: contains datablock(s) I. DOI: 10.1107/S1600536812023458/wn2476Isup2.hkl

Supplementary material file. DOI: 10.1107/S1600536812023458/wn2476Isup3.cml

Additional supplementary materials:  crystallographic information; 3D view; checkCIF report

## Figures and Tables

**Table 1:** Hydrogen-bond geometry (Å, °)

*D*—H⋯*A*	*D*—H	H⋯*A*	*D*⋯*A*	*D*—H⋯*A*
N11*A*—H11*A*⋯O21	0.87	2.01	2.866 (4)	168
N11*A*—H12*A*⋯O12^i^	0.90	1.90	2.801 (5)	180
N11*A*—H13*A*⋯O22^ii^	0.88	1.94	2.816 (4)	175
N11*B*—H11*B*⋯O11^i^	0.88	1.96	2.823 (4)	167
N11*B*—H12*B*⋯O21	0.96	1.85	2.770 (4)	160
N11*B*—H13*B*⋯O22^iii^	0.82	1.99	2.803 (4)	172
O1*W*—H11*W*⋯O11^i^	0.85	1.94	2.789 (4)	179
O1*W*—H12*W*⋯O12	0.76	2.28	2.980 (4)	154

Symmetry codes: (i) 
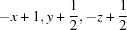
; (ii) 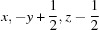; (iii) 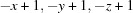.

**Fig. 1.**
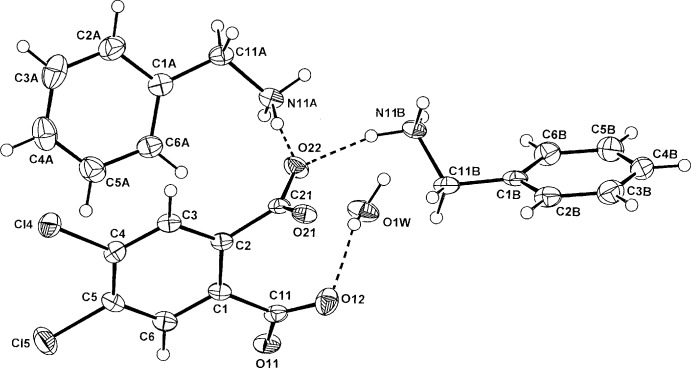
Structure of the two cations (A and B), the dianion and the water molecule of solvation in the asymmetric unit of the title salt, with the inter-species hydrogen bonds shown as dashed lines. Non-H atoms are shown as 40% probability displacement ellipsoids. Hydrogen atoms are shown as spheres of arbitrary radius.

**Fig. 2.**
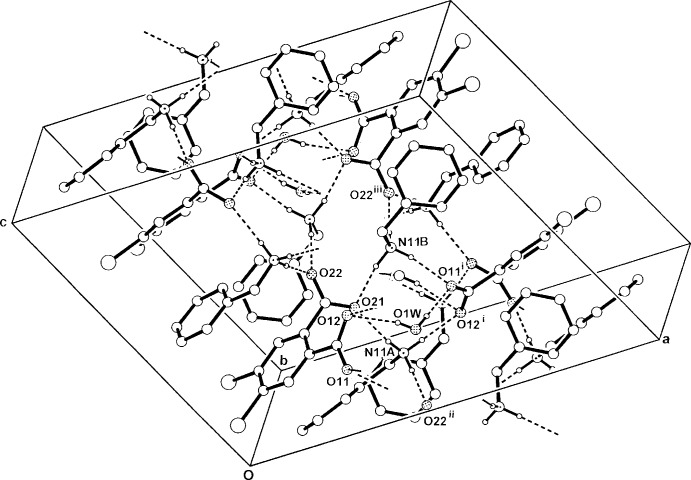
A perspective view of part of the two-dimensional duplex-sheet structure in the unit cell, showing hydrogen-bonding associations as dashed lines. Non-associative H-atoms are omitted. For symmetry codes, see Table 1.

## supplementary crystallographic information

### Comment

4,5-Dichlorophthalic acid (DCPA) most commonly forms 1:1 salts with the Lewis bases, often giving low-dimensional hydrogen-bonded structures (Mattes & Dorau, 1986; Smith *et al.*, 2008). The structures of the 2:1 Lewis base salts of DCPA are less common; the bis(4-ethylaminium) salt (Büyükgüngör & Odabaşoğlu, 2007) and the bis(guanidinium) salt (Smith & Wermuth, 2011) are among these while the DCPA dianion is also found in the ethylenediaminium salt (Smith & Wermuth, 2010). However, our 1:1 stoichiometric reaction of DCPA with benzylamine gave unexpectedly a 2:1 salt 2(C_7_H_10_N^+^) C_8_H_2_Cl_2_O_4_^2-^. H_2_O, the title compound, and the structure is reported here.

In this structure (Fig. 1), the two benzylaminium cations (*A* and *B*) have very different conformations, one being essentially planar the other having the side-chain rotated out of the benzene plane [minimum ring to side-chain C—C—C—N torsion angles = -3.6 (6)° (*A*) and 50.1 (5)° *B*]. In the 4,5-dichlorophthalate dianion the carboxyl groups make dihedral angles of 23.0 (2) and 76.5 (2)° with the benzene ring, corresponding to torsion angles C1—C2—C21—O21 and C2—C1—C11—O11 of -157.7 (4) and 78.1 (5)°. Aminium N—H···O and water O—H···O hydrogen-bonding associations with carboxyl O-atom acceptors (Table 1) give a two-dimensional duplex-sheet structure which extends along the (011) plane (Fig. 2). Weak π–π interactions are also present between the benzene ring of the DCPA dianion and one of the cation rings (*A*) [minimum ring centroid separation, 2.749 (3) Å].

### Experimental

The title compound was synthesized by heating together, for 10 min under reflux, 1 mmol quantities of 4,5-dichlorophthalic acid and benzylamine in 50 ml of methanol. Partial evaporation of the solvent gave colourless crystalline plates of the title compound from which a specimen was cleaved for the X-ray analysis.

### Refinement

Hydrogen atoms potentially involved in hydrogen-bonding interactions were located by difference methods and their positional and isotropic displacement parameters were initially refined. However, in the final refinement cycles these were set invariant with the displacement parameters riding on the parent atom [with *U*_iso_(H) = 1.2*U*_eq_(N) or 1.5*U*_eq_(O)]. Other H atoms were included at calculated positions [C—H (aromatic) = 0.93 Å or C—H (methylene) = 0.97 Å] and allowed to ride, with *U*_iso_(H) = 1.2*U*_eq_(C).

### Crystal data

**Table:**

2C_7_H_10_N^+^·C_8_H_2_Cl_2_O_4_^2^^−^·H_2_O	*F*(000) = 976
*M**_r_* = 467.33	*D*_x_ = 1.418 Mg m^−^^3^
Monoclinic, *P*2_1_/*c*	Mo *K*α radiation, λ = 0.71073 Å
Hall symbol: -P 2ybc	Cell parameters from 6279 reflections
*a* = 17.3005 (16) Å	θ = 3.2–28.7°
*b* = 10.0084 (7) Å	µ = 0.33 mm^−^^1^
*c* = 13.6990 (12) Å	*T* = 200 K
β = 112.641 (11)°	Plate, colourless
*V* = 2189.2 (4) Å^3^	0.33 × 0.22 × 0.12 mm
*Z* = 4	

### Data collection

**Table:**

Oxford Diffraction Gemini-S CCD-detector diffractometer	3842 independent reflections
Radiation source: Enhance (Mo) X-ray source	3019 reflections with *I* > 2σ(*I*)
Graphite monochromator	*R*_int_ = 0.045
Detector resolution: 16.077 pixels mm^-1^	θ_max_ = 25.0°, θ_min_ = 3.2°
ω scans	*h* = −20→20
Absorption correction: multi-scan (*CrysAlis PRO*; Oxford Diffraction, 2010)	*k* = −11→11
*T*_min_ = 0.86, *T*_max_ = 0.98	*l* = −16→16
12311 measured reflections	

### Refinement

**Table:**

Refinement on *F*^2^	Primary atom site location: structure-invariant direct methods
Least-squares matrix: full	Secondary atom site location: difference Fourier map
*R*[*F*^2^ > 2σ(*F*^2^)] = 0.068	Hydrogen site location: inferred from neighbouring sites
*wR*(*F*^2^) = 0.189	H-atom parameters constrained
*S* = 1.19	*w* = 1/[σ^2^(*F*_o_^2^) + (0.0794*P*)^2^ + 3.4869*P*] where *P* = (*F*_o_^2^ + 2*F*_c_^2^)/3
3842 reflections	(Δ/σ)_max_ = 0.001
312 parameters	Δρ_max_ = 0.79 e Å^−^^3^
0 restraints	Δρ_min_ = −0.37 e Å^−^^3^

### Special details

**Table:**

Geometry. Bond distances, angles *etc*. have been calculated using the rounded fractional coordinates. All su's are estimated from the variances of the (full) variance-covariance matrix. The cell e.s.d.'s are taken into account in the estimation of distances, angles and torsion angles
Refinement. Refinement of *F*^2^ against ALL reflections. The weighted *R*-factor *wR* and goodness of fit *S* are based on *F*^2^, conventional *R*-factors *R* are based on *F*, with *F* set to zero for negative *F*^2^. The threshold expression of *F*^2^ > σ(*F*^2^) is used only for calculating *R*-factors(gt) *etc*. and is not relevant to the choice of reflections for refinement. *R*-factors based on *F*^2^ are statistically about twice as large as those based on *F*, and *R*- factors based on ALL data will be even larger.

### Fractional atomic coordinates and isotropic or equivalent isotropic displacement parameters (Å^2^)

**Table:**

	*x*	*y*	*z*	*U*_iso_*/*U*_eq_	
N11A	0.4026 (2)	0.4021 (3)	0.0987 (2)	0.0293 (11)	
C1A	0.2474 (3)	0.4407 (4)	0.0233 (3)	0.0307 (11)	
C2A	0.1790 (3)	0.5252 (5)	−0.0185 (4)	0.0460 (17)	
C3A	0.0984 (3)	0.4801 (6)	−0.0447 (4)	0.0571 (19)	
C4A	0.0838 (3)	0.3462 (6)	−0.0318 (4)	0.0515 (19)	
C5A	0.1508 (3)	0.2619 (5)	0.0077 (4)	0.0443 (16)	
C6A	0.2319 (3)	0.3065 (4)	0.0357 (3)	0.0353 (14)	
C11A	0.3333 (3)	0.4980 (4)	0.0506 (3)	0.0322 (12)	
N11B	0.5631 (2)	0.4462 (3)	0.4310 (2)	0.0270 (10)	
C1B	0.6924 (3)	0.3655 (4)	0.5777 (3)	0.0295 (11)	
C2B	0.7238 (3)	0.3664 (4)	0.6877 (3)	0.0378 (14)	
C3B	0.8081 (3)	0.3812 (5)	0.7454 (4)	0.0446 (16)	
C4B	0.8630 (3)	0.3963 (4)	0.6953 (4)	0.0431 (16)	
C5B	0.8322 (3)	0.3971 (5)	0.5861 (4)	0.0443 (17)	
C6B	0.7479 (3)	0.3822 (5)	0.5275 (4)	0.0392 (16)	
C11B	0.6004 (3)	0.3454 (4)	0.5161 (3)	0.0329 (12)	
Cl4	0.05397 (7)	0.26123 (11)	0.21573 (9)	0.0404 (4)	
Cl5	0.06311 (7)	−0.04167 (12)	0.15221 (10)	0.0496 (4)	
O11	0.38460 (19)	−0.0979 (3)	0.2378 (2)	0.0332 (9)	
O12	0.45046 (18)	0.0453 (3)	0.3688 (2)	0.0394 (10)	
O21	0.40882 (18)	0.3398 (3)	0.3058 (2)	0.0353 (9)	
O22	0.39728 (17)	0.3096 (3)	0.4609 (2)	0.0285 (8)	
C1	0.3039 (2)	0.0669 (4)	0.2786 (3)	0.0243 (11)	
C2	0.2997 (2)	0.1997 (4)	0.3108 (3)	0.0232 (11)	
C3	0.2221 (2)	0.2560 (4)	0.2924 (3)	0.0259 (11)	
C4	0.1492 (2)	0.1842 (4)	0.2424 (3)	0.0283 (12)	
C5	0.1534 (3)	0.0524 (4)	0.2126 (3)	0.0302 (12)	
C6	0.2302 (3)	−0.0051 (4)	0.2304 (3)	0.0293 (12)	
C11	0.3866 (3)	0.0005 (4)	0.2974 (3)	0.0278 (12)	
C21	0.3755 (2)	0.2872 (4)	0.3636 (3)	0.0240 (11)	
O1W	0.5248 (2)	0.1641 (3)	0.2253 (2)	0.0437 (11)	
H2A	0.18780	0.61480	−0.02910	0.0550*	
H3A	0.05360	0.53930	−0.07100	0.0690*	
H4A	0.02950	0.31450	−0.04970	0.0620*	
H5A	0.14150	0.17180	0.01590	0.0530*	
H6A	0.27630	0.24680	0.06290	0.0420*	
H11A	0.40280	0.37170	0.15800	0.0350*	
H12A	0.44980	0.44770	0.10900	0.0350*	
H13A	0.40080	0.33970	0.05300	0.0350*	
H14A	0.33700	0.53380	−0.01330	0.0390*	
H15A	0.34070	0.57180	0.09930	0.0390*	
H2B	0.68730	0.35690	0.72240	0.0450*	
H3B	0.82830	0.38090	0.81890	0.0540*	
H4B	0.92010	0.40590	0.73450	0.0520*	
H5B	0.86890	0.40780	0.55180	0.0530*	
H6B	0.72790	0.38340	0.45400	0.0470*	
H11B	0.58300	0.44510	0.38110	0.0320*	
H12B	0.50500	0.42810	0.39110	0.0320*	
H13B	0.57000	0.52010	0.45910	0.0320*	
H14B	0.59160	0.25700	0.48470	0.0390*	
H15B	0.57160	0.34930	0.56430	0.0390*	
H3	0.21900	0.34330	0.31390	0.0310*	
H6	0.23260	−0.09300	0.20990	0.0350*	
H11W	0.55210	0.23660	0.23670	0.0650*	
H12W	0.50510	0.15860	0.26570	0.0650*	

### Atomic displacement parameters (Å^2^)

**Table:**

	*U*^11^	*U*^22^	*U*^33^	*U*^12^	*U*^13^	*U*^23^
N11A	0.037 (2)	0.0257 (18)	0.0298 (17)	−0.0037 (15)	0.0179 (15)	−0.0026 (14)
C1A	0.040 (2)	0.030 (2)	0.0254 (19)	0.0025 (19)	0.0164 (18)	−0.0033 (16)
C2A	0.048 (3)	0.036 (3)	0.052 (3)	0.008 (2)	0.017 (2)	0.002 (2)
C3A	0.040 (3)	0.069 (4)	0.058 (3)	0.018 (3)	0.014 (2)	−0.002 (3)
C4A	0.039 (3)	0.079 (4)	0.040 (3)	−0.008 (3)	0.019 (2)	−0.004 (3)
C5A	0.045 (3)	0.050 (3)	0.041 (2)	−0.009 (2)	0.020 (2)	0.003 (2)
C6A	0.043 (3)	0.036 (2)	0.031 (2)	0.002 (2)	0.0188 (19)	0.0054 (18)
C11A	0.041 (2)	0.023 (2)	0.033 (2)	0.0014 (18)	0.0148 (18)	−0.0012 (17)
N11B	0.0371 (19)	0.0191 (16)	0.0293 (16)	−0.0034 (14)	0.0179 (15)	−0.0041 (13)
C1B	0.038 (2)	0.0147 (18)	0.041 (2)	0.0021 (17)	0.0210 (19)	0.0040 (16)
C2B	0.045 (3)	0.031 (2)	0.042 (2)	0.002 (2)	0.022 (2)	0.0070 (19)
C3B	0.049 (3)	0.040 (3)	0.041 (2)	0.002 (2)	0.013 (2)	0.004 (2)
C4B	0.034 (3)	0.027 (2)	0.064 (3)	0.0046 (19)	0.014 (2)	−0.001 (2)
C5B	0.041 (3)	0.039 (3)	0.062 (3)	0.004 (2)	0.030 (2)	0.003 (2)
C6B	0.042 (3)	0.040 (3)	0.041 (2)	0.004 (2)	0.022 (2)	0.005 (2)
C11B	0.041 (2)	0.021 (2)	0.043 (2)	0.0023 (18)	0.023 (2)	0.0063 (17)
Cl4	0.0307 (6)	0.0397 (6)	0.0530 (7)	0.0075 (5)	0.0185 (5)	0.0053 (5)
Cl5	0.0350 (6)	0.0422 (7)	0.0676 (8)	−0.0120 (5)	0.0155 (6)	−0.0114 (6)
O11	0.0445 (18)	0.0204 (14)	0.0431 (16)	0.0051 (13)	0.0262 (14)	−0.0038 (12)
O12	0.0333 (17)	0.0378 (17)	0.0430 (17)	0.0084 (14)	0.0103 (14)	−0.0079 (14)
O21	0.0378 (17)	0.0388 (17)	0.0327 (15)	−0.0107 (14)	0.0173 (13)	0.0040 (13)
O22	0.0405 (16)	0.0210 (14)	0.0276 (14)	−0.0035 (12)	0.0171 (12)	−0.0039 (11)
C1	0.035 (2)	0.0204 (19)	0.0226 (18)	−0.0011 (16)	0.0169 (16)	0.0002 (15)
C2	0.031 (2)	0.0222 (19)	0.0221 (17)	0.0015 (16)	0.0164 (16)	0.0029 (15)
C3	0.036 (2)	0.0210 (19)	0.0255 (19)	0.0025 (17)	0.0173 (17)	−0.0015 (15)
C4	0.030 (2)	0.032 (2)	0.029 (2)	0.0059 (18)	0.0180 (17)	0.0040 (17)
C5	0.035 (2)	0.025 (2)	0.032 (2)	−0.0073 (18)	0.0146 (18)	−0.0015 (16)
C6	0.038 (2)	0.024 (2)	0.031 (2)	−0.0005 (18)	0.0189 (18)	−0.0018 (16)
C11	0.034 (2)	0.023 (2)	0.032 (2)	0.0036 (17)	0.0188 (18)	0.0075 (17)
C21	0.029 (2)	0.0151 (18)	0.031 (2)	0.0028 (15)	0.0150 (17)	0.0032 (15)
O1W	0.059 (2)	0.0254 (16)	0.0577 (19)	−0.0065 (15)	0.0345 (17)	−0.0072 (14)

### Geometric parameters (Å, º)

**Table:**

Cl4—C4	1.726 (4)	C6A—H6A	0.9300
Cl5—C5	1.737 (5)	C11A—H15A	0.9700
O11—C11	1.271 (5)	C11A—H14A	0.9700
O12—C11	1.244 (5)	C1B—C2B	1.391 (5)
O21—C21	1.260 (5)	C1B—C11B	1.501 (7)
O22—C21	1.258 (5)	C1B—C6B	1.390 (7)
O1W—H11W	0.8500	C2B—C3B	1.373 (7)
O1W—H12W	0.7600	C3B—C4B	1.378 (8)
N11A—C11A	1.478 (6)	C4B—C5B	1.381 (7)
N11A—H13A	0.8800	C5B—C6B	1.376 (8)
N11A—H11A	0.8700	C2B—H2B	0.9300
N11A—H12A	0.9000	C3B—H3B	0.9300
N11B—C11B	1.488 (5)	C4B—H4B	0.9300
N11B—H12B	0.9600	C5B—H5B	0.9300
N11B—H13B	0.8200	C6B—H6B	0.9300
N11B—H11B	0.8800	C11B—H15B	0.9700
C1A—C6A	1.393 (6)	C11B—H14B	0.9700
C1A—C2A	1.386 (7)	C1—C11	1.507 (6)
C1A—C11A	1.500 (7)	C1—C2	1.411 (6)
C2A—C3A	1.375 (8)	C1—C6	1.390 (6)
C3A—C4A	1.388 (8)	C2—C21	1.510 (5)
C4A—C5A	1.366 (8)	C2—C3	1.387 (5)
C5A—C6A	1.379 (8)	C3—C4	1.382 (5)
C2A—H2A	0.9300	C4—C5	1.391 (6)
C3A—H3A	0.9300	C5—C6	1.381 (7)
C4A—H4A	0.9300	C3—H3	0.9300
C5A—H5A	0.9300	C6—H6	0.9300
			
H11W—O1W—H12W	108.00	C4B—C5B—C6B	120.8 (5)
C11A—N11A—H13A	110.00	C1B—C6B—C5B	120.2 (5)
C11A—N11A—H11A	111.00	N11B—C11B—C1B	113.3 (4)
H12A—N11A—H13A	105.00	C1B—C2B—H2B	120.00
H11A—N11A—H13A	114.00	C3B—C2B—H2B	120.00
H11A—N11A—H12A	111.00	C4B—C3B—H3B	120.00
C11A—N11A—H12A	106.00	C2B—C3B—H3B	120.00
C11B—N11B—H11B	115.00	C3B—C4B—H4B	120.00
H12B—N11B—H13B	112.00	C5B—C4B—H4B	120.00
C11B—N11B—H13B	108.00	C4B—C5B—H5B	120.00
H11B—N11B—H13B	110.00	C6B—C5B—H5B	120.00
C11B—N11B—H12B	111.00	C5B—C6B—H6B	120.00
H11B—N11B—H12B	101.00	C1B—C6B—H6B	120.00
C6A—C1A—C11A	123.9 (4)	N11B—C11B—H15B	109.00
C2A—C1A—C11A	118.4 (4)	C1B—C11B—H14B	109.00
C2A—C1A—C6A	117.7 (5)	C1B—C11B—H15B	109.00
C1A—C2A—C3A	121.7 (5)	H14B—C11B—H15B	108.00
C2A—C3A—C4A	120.0 (5)	N11B—C11B—H14B	109.00
C3A—C4A—C5A	118.6 (5)	C2—C1—C6	119.2 (4)
C4A—C5A—C6A	121.9 (5)	C2—C1—C11	121.4 (3)
C1A—C6A—C5A	120.1 (4)	C6—C1—C11	119.3 (4)
N11A—C11A—C1A	114.8 (3)	C1—C2—C21	123.8 (3)
C3A—C2A—H2A	119.00	C3—C2—C21	116.9 (3)
C1A—C2A—H2A	119.00	C1—C2—C3	119.3 (4)
C2A—C3A—H3A	120.00	C2—C3—C4	120.9 (4)
C4A—C3A—H3A	120.00	Cl4—C4—C3	119.2 (3)
C3A—C4A—H4A	121.00	Cl4—C4—C5	121.0 (3)
C5A—C4A—H4A	121.00	C3—C4—C5	119.8 (4)
C4A—C5A—H5A	119.00	Cl5—C5—C6	119.1 (3)
C6A—C5A—H5A	119.00	C4—C5—C6	119.9 (4)
C1A—C6A—H6A	120.00	Cl5—C5—C4	121.0 (4)
C5A—C6A—H6A	120.00	C1—C6—C5	120.8 (4)
H14A—C11A—H15A	108.00	O11—C11—C1	116.3 (4)
N11A—C11A—H14A	109.00	O12—C11—C1	118.2 (4)
N11A—C11A—H15A	109.00	O11—C11—O12	125.5 (5)
C1A—C11A—H14A	109.00	O21—C21—C2	117.7 (3)
C1A—C11A—H15A	109.00	O22—C21—C2	117.5 (3)
C2B—C1B—C6B	118.6 (5)	O21—C21—O22	124.7 (4)
C2B—C1B—C11B	119.8 (4)	C2—C3—H3	120.00
C6B—C1B—C11B	121.6 (4)	C4—C3—H3	119.00
C1B—C2B—C3B	120.6 (5)	C1—C6—H6	120.00
C2B—C3B—C4B	120.5 (5)	C5—C6—H6	120.00
C3B—C4B—C5B	119.2 (5)		
			
C6A—C1A—C2A—C3A	1.6 (7)	C11—C1—C2—C3	−179.5 (4)
C11A—C1A—C2A—C3A	−179.5 (4)	C11—C1—C2—C21	1.4 (6)
C2A—C1A—C6A—C5A	−0.6 (6)	C2—C1—C6—C5	0.9 (6)
C11A—C1A—C6A—C5A	−179.4 (4)	C11—C1—C6—C5	179.6 (4)
C2A—C1A—C11A—N11A	177.6 (4)	C2—C1—C11—O11	−157.7 (4)
C6A—C1A—C11A—N11A	−3.6 (6)	C2—C1—C11—O12	22.4 (6)
C1A—C2A—C3A—C4A	−1.5 (8)	C6—C1—C11—O11	23.6 (6)
C2A—C3A—C4A—C5A	0.4 (8)	C6—C1—C11—O12	−156.3 (4)
C3A—C4A—C5A—C6A	0.6 (8)	C1—C2—C3—C4	−0.5 (6)
C4A—C5A—C6A—C1A	−0.5 (7)	C21—C2—C3—C4	178.6 (3)
C6B—C1B—C2B—C3B	−1.1 (6)	C1—C2—C21—O21	78.1 (5)
C11B—C1B—C2B—C3B	178.3 (4)	C1—C2—C21—O22	−106.7 (5)
C2B—C1B—C6B—C5B	1.0 (7)	C3—C2—C21—O21	−101.0 (4)
C11B—C1B—C6B—C5B	−178.4 (4)	C3—C2—C21—O22	74.2 (5)
C2B—C1B—C11B—N11B	130.6 (4)	C2—C3—C4—Cl4	−177.0 (3)
C6B—C1B—C11B—N11B	−50.1 (5)	C2—C3—C4—C5	1.8 (6)
C1B—C2B—C3B—C4B	0.4 (7)	Cl4—C4—C5—Cl5	−2.6 (5)
C2B—C3B—C4B—C5B	0.3 (7)	Cl4—C4—C5—C6	177.0 (3)
C3B—C4B—C5B—C6B	−0.4 (7)	C3—C4—C5—Cl5	178.6 (3)
C4B—C5B—C6B—C1B	−0.3 (7)	C3—C4—C5—C6	−1.8 (6)
C6—C1—C2—C3	−0.8 (6)	Cl5—C5—C6—C1	−180.0 (3)
C6—C1—C2—C21	−179.9 (4)	C4—C5—C6—C1	0.4 (6)

### Hydrogen-bond geometry (Å, º)

**Table:**

*D*—H···*A*	*D*—H	H···*A*	*D*···*A*	*D*—H···*A*
N11*A*—H11*A*···O21	0.87	2.01	2.866 (4)	168
N11*A*—H12*A*···O12^i^	0.90	1.90	2.801 (5)	180
N11*A*—H13*A*···O22^ii^	0.88	1.94	2.816 (4)	175
N11*B*—H11*B*···O11^i^	0.88	1.96	2.823 (4)	167
N11*B*—H12*B*···O21	0.96	1.85	2.770 (4)	160
N11*B*—H13*B*···O22^iii^	0.82	1.99	2.803 (4)	172
O1*W*—H11*W*···O11^i^	0.85	1.94	2.789 (4)	179
O1*W*—H12*W*···O12	0.76	2.28	2.980 (4)	154

Symmetry codes: (i) −*x*+1, *y*+1/2, −*z*+1/2; (ii) *x*, −*y*+1/2, *z*−1/2; (iii) −*x*+1, −*y*+1, −*z*+1.
